# Complexity and Demographic Explanations of Cumulative Culture

**DOI:** 10.1371/journal.pone.0102543

**Published:** 2014-07-21

**Authors:** Adrien Querbes, Krist Vaesen, Wybo Houkes

**Affiliations:** 1 Department of Social and Decision Sciences (SDS), Carnegie Mellon University (CMU), Pittsburgh, Pennsylvania, United States of America; 2 School of Innovation Sciences, Eindhoven University of Technology, Eindhoven, The Netherlands; Universidade do Algarve, Portugal

## Abstract

Formal models have linked prehistoric and historical instances of technological change (e.g., the Upper Paleolithic transition, cultural loss in Holocene Tasmania, scientific progress since the late nineteenth century) to demographic change. According to these models, cumulation of technological complexity is inhibited by decreasing— while favoured by increasing—population levels. Here we show that these findings are contingent on how complexity is defined: demography plays a much more limited role in sustaining cumulative culture in case formal models deploy Herbert Simon's definition of complexity rather than the particular definitions of complexity hitherto assumed. Given that currently available empirical evidence doesn't afford discriminating proper from improper definitions of complexity, our robustness analyses put into question the force of recent demographic explanations of particular episodes of cultural change.

## Introduction

One of the key insights of cultural evolutionary theory is that cumulative culture crucially depends on demography [1]. Indeed, a wide variety of models of cultural transmission has reproduced the result that changes in population size may drive cultural change: increases in the former favour cumulation, while decreases may occasion cultural loss ([2]–[7], but see [8]). These model-theoretical findings are used to explain particular cultural transitions, e.g., the loss of culture in Holocene Tasmania, the Upper Paleolithic transition, or the growth of scientific knowledge since the Industrial Revolution.

Interestingly, although said models express cumulation in terms of complexity increases, they differ considerably in how they construe the latter term (i.e. complexity). Characterizations have been given in terms of change in fitness [2]; in terms of transmission inaccuracy [3]–[6]; and in terms of the number of elements a cultural trait consists of [7]. This may be an asset rather than a drawback: if there is convergence of results, demographic change may be offered as a possible explanation of a broad suite of patterns of cultural change, viz. all patterns that can be plausibly construed as cumulative under one of the various characterizations on offer. This is especially pertinent in case empirical evidence is not sufficiently abundant to prefer one particular construal of cultural change.

Consider for instance the Upper Paleolithic transition, which according to Powell et al [4] is characterized by "substantial increase in technological and cultural complexity, including the first consistent presence of symbolic behavior, such as abstract and realistic art and body decoration […], systematically produced microlithic stone tools […], functional and ritual bone, antler, and ivory artifacts, grinding and pounding stone tools, improved hunting and trapping technology […], an increase in the long-distance transfer of raw materials, and musical instruments." As intuitively plausible as this may seem, it still needs to be established that the Late Pleistocene cultural complexity referred to by Powell and colleagues really is adequately captured by the characterization of complexity assumed in current models (including their own). For example, does the emergence and consistent presence of symbolic behaviour demonstrate that cultural skills become more complex in the sense of becoming harder to transmit faithfully? That is, was Upper Paleolithic symbolic behaviour actually more error-prone than previous (non-symbolic) behaviour? Or is this transition better understood in terms of an increase in the number of elements cultural behaviours encompassed, with symbolic behaviours encompassing more elements than previous (non-symbolic) behaviours? Does the same apply for long-distance transfer of raw materials? For hunting technologies? Currently available evidence does not afford conclusive answers to these questions; arguably, the answers are underdetermined by any conceivable evidence. In this epistemic situation, the diversity of characterizations of complexity—and of model assumptions more generally—may save the day: the larger the set of intuitively plausible definitions of complexity in the family of demographic models, the more likely it is that at least one empirically valid characterization of the considered pattern of change is included in the set. That is, the more robust the relation between population size and cumulative cultural change is under variations of characterizations of cumulation and of modelling assumptions, the more credible or widely applicable are demographic explanations of cultural change. Conversely, if the dependence fails to obtain under some characterizations or auxiliary assumptions, an episode of cultural change can only be justifiably attributed to demographic change in case empirical evidence speaks against these unfavourable characterizations or assumptions.

In this paper, we offer a cultural-evolutionary model that is based on yet another characterization of complexity, and we examine whether it can be safely added to the family of models that show a relation between cultural and demographic change. If it can, the dependence of cumulative culture on demography would stand even firmer. If it can not, patterns of cultural change that are cumulative in our newly introduced terms have not yet been shown to be susceptible to demographic explanation.

**Figure 1:**
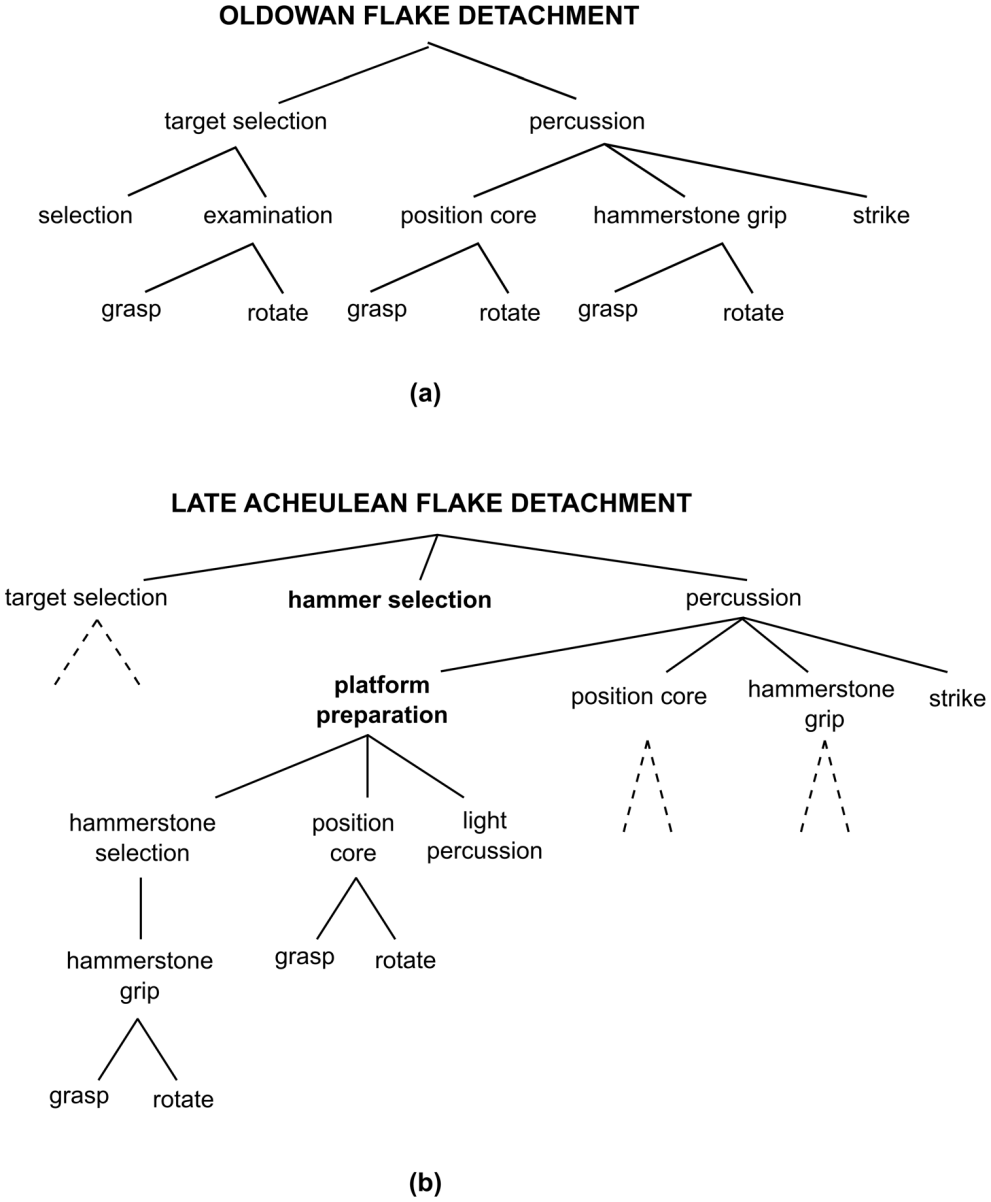
Action hierarchies for (a) Oldowan flake detachment; (b) Late Acheulean flake detachment (redrawn detail from [10]). Lines connect subordinate elements with the superordinate element they instantiate. In (b), dashed lines indicate action chunks which are identical to those defined in (a).

We characterize cumulation, like some existing contributions to the literature, in terms of increasing complexity. Yet rather than characterizing transmission accuracy or the sheer number of elements in a cultural trait, we follow Herbert Simon [9] in taking complexity to consist in the density of *interaction* between the elements of a trait: cultural change is cumulative in case the transmission of cultural traits sustains ever more intricate interdependencies between the components or elements of these traits. To illustrate the plausibility of the assumption, consider the production of early stone tools. Figure 1 presents action hierarchies for Oldowan and Late Acheulean flake detachment (after [10]; see also references therein). The latter is more complex than the former not primarily because it has more constitutive elements, but rather because these elements are organized in a more elaborate hierarchical structure that comprises more nested levels: the addition of platform preparation to the superordinate goal of percussion in Late Acheulean flake detachment introduces four nested levels, so that the method contains six nested levels in total (versus four in Oldowan flake detachment). The success of the superordinate level (i.e. percussion) is thus contingent on four elements (rather than three, as in Oldowan production), namely position core, hammerstone grip, strike *plus* platform preparation, and the success of the latter is itself contingent on the interplay of a whole set of lower-level actions. Action hierarchies for multi-component blade technologies would be even more intricate. Here the success of percussion would, for example, depend on stringent selection/importation of raw materials [11]–[13] and on the properties of other components (such as the haft). The more intricately these elements are interrelated, the more difficult it becomes to predict how changes in one place will affect elements elsewhere in the hierarchy. Even a very small error introduced during transmission in one element may have profound repercussions on the performance of other elements, and thus on success overall (which in turn makes it difficult to predict expected changes through time, see [14]). Therefore, in cases like these, complexity defined as transmission inaccuracy and complexity as defined by Simon (let us call it S-complexity) need to be carefully distinguished.

The literature on cultural evolution has not overlooked S-complexity. Most importantly, the intricate interaction between the material components of technologies has been offered as evidence that cumulative cultural changes can not have been the result of individual learning: "[Several technologies] are very complicated artifacts with multiple interacting parts made of many different materials. (…) Determining the best design is, in effect, a high dimensional optimization problem that is usually beyond individual cognitive capacities…" [15]. Similarly, Mesoudi and O'Brien have introduced a two-peaked fitness landscape in their experimental study of the transmission of projectile-point design [16]. Despite this supporting role of S-complexity in cultural-evolutionary theory, it has not yet been implemented in cultural-evolutionary models.

There is at least one reason to expect that doing so has strong, and negative, repercussions for demographic explanations. The intuition behind the dependence of cumulative cultural change on demography is strength in numbers: larger populations can sustain a more complex culture simply because they are more likely to contain individuals whose cultural traits are at least as good as those of the individuals they imitated from previous generations. However, under the assumption that mentor selection is imperfect, populations as a whole profit from such outstanding individuals only insofar as these can be detected by others as suitable objects of imitation—and here larger populations are clearly at a *dis*advantage. At high levels of S-complexity, this disadvantage may become insurmountable. For under these conditions, only very sizable populations will contain any individuals who have been able to avoid the slight transmission inaccuracy in cultural transmission that would have interfered with overall success of the complex cultural trait. These individuals might not in turn be able to transmit their traits, however, since potential students might be unable to find them in a population of this size.

## Materials and Methods

We devised a model with successive generations of agents, each of whom learns from a parent of the previous generation through oblique, pay-off based transmission—much as in [3]–[6], [8]. Adjustments to these models, required to implement S-complexity, were based on Stuart Kauffman's so-called *NK*-logic [17]. In line with Herbert Simon's proposal, 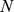 here stands for the number of components, whereas 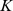 expresses the level of interaction between components. Let us explain the model in more detail.

The agent-based model (implemented in Netlogo, code available from the authors) contains a population of 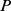 agents, each of whom exhibits a variant of a cultural trait (say, a technology or technological skill). The configuration of any variant is given by a binary string of 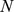 elements. For example, if 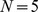, the string *01101* would refer to a variant which differs in the second element from a variant characterized by *00101*. We follow Kauffman in assuming that each element can be only in two states (*0* or *1*). Although it is in principle possible to extend the model assuming any number of possible states, working with binary values is intuitive enough. For example, consider percussion in Late Acheulean flake detachment (see Figure 1b), which consists of four elements: platform preparation, positioning of the core, holding the hammerstone, and striking. For each of these actions we could assign a *1* when the action is executed in one way, and a *0* when executed in another way. Percussion on a prepared platform would then be represented by the string *1111*, percussion on the ground by the string *0111*, and so forth. Evidently, one could increase the level of precision by adding more elements. For instance, one could characterize platform preparation with a three-element string, stating values for hammerstone selection, positioning of the core and light percussion.

To any variant is assigned a skill or fitness value, 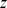, which is defined as the average of the *contributing values* of each of the 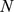 individual elements; the contributing value of each element is in turn determined by its own state (*0* or *1*), and by the state of 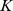 other elements. In case of 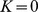, changing the state of one element (e.g., from *0* to *1*), will only affect the value of the element itself. In case of 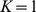, the contributing value of an element may change directly or as a result of a change in the single other element influencing it. The interaction parameter 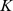 can be any natural number between *0* and 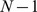.

By changing the parameters *N* and *K* we can tune the complexity of cultural traits. The least complex trait is one for which 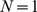 and, per definition, 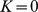. Complexity increases with increasing 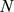; further, for any 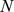, maximal complexity is obtained when 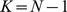. Under the latter circumstances, a change in one element affects the fitness of all other elements. In line with previous research [3]–[6], we use transmission inaccuracy as an additional measure of complexity. However, whereas transmission inaccuracy has been previously expressed only as the magnitude of the error naïve individuals make, in our paper it is determined by a copy error rate 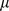, i.e. a number expressing the *probability* that a transmission error will be made, where an error consists in unfaithful replication of an element of the copied sequence. Multiple errors (i.e. elements changing states) may occur in the same transmission attempt. The *impact* of these errors can, like 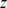, be expressed as a real number between 0 and 1.

**Figure 2:**
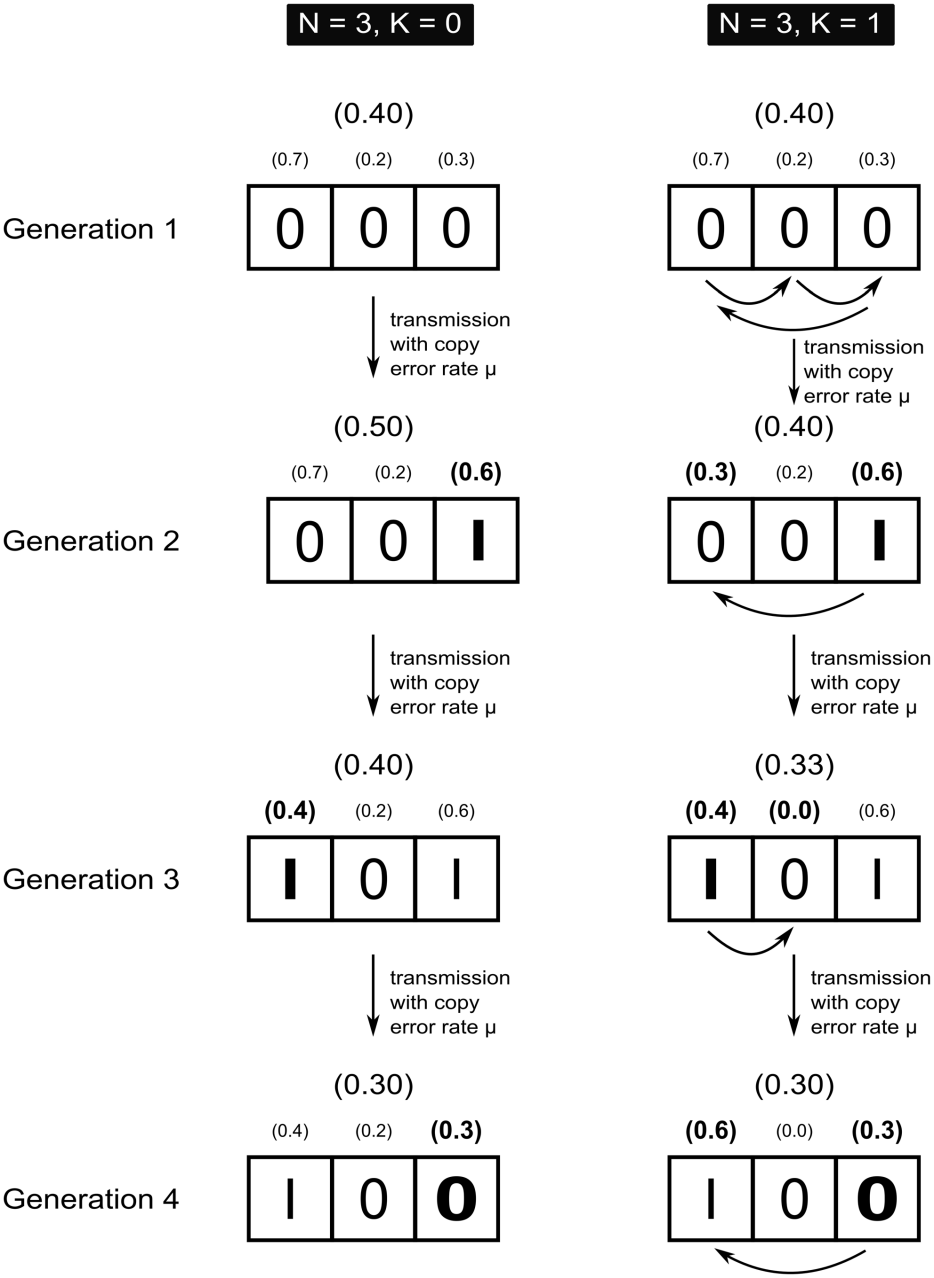
Illustration of the NK-logic. Left, 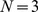, 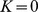: Copy errors only affect the fitness of the element in question. Right, 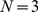, 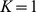: Copy errors affect the fitness of the element in question *and* the fitness of one (given 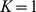) other element. The direction of these interactions is represented by the arrows underneath the string of Generation 1.

Figure 2 illustrates the *NK*-logic, as well as transmission over successive generations, for a population size 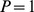. On the left is represented a trait with three elements (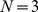) without interdependencies between elements (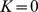). The variant of the trait in Generation 1 is *000*, with an average fitness of 0.4. Now the offspring in Generation 2 will try to imitate the cultural parent in Generation 1. Note that in case 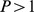, offspring will get the opportunity to select a cultural parent, but more on this below.

Imitation happens with copy error rate 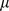, which is in our model defined as the probability of an element changing state in a transmission attempt. So for 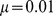, any element has a 1% chance of changing state (from *0* to *1* or from *1* to *0*). In the worked-out example, we assume deterministic inaccuracy for the sake of clarity: in each generation, exactly one element changes state. From Generation 1 to Generation 2, the third element is copied inaccurately, and thus receives a new contributing value (from 0.3 to 0.6, the magnitude of the error being 0.3). Since 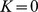, this mutation does not affect any other element in the string.

Compare this with the right-hand side of Figure 2, where 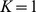, which means that each element interacts with one other element in the string. In this example, these interactions are one-sided influences, of the first element on the second element, of the second on the third, and of the third element on the first. The interactions in this example are regular, but this need not to be the case. For if, for example, 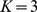, an element has three links coming in, but an *average* of 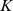 links coming out. Now, for example, if the third element changes state, as when going from Generation 1 to Generation 2, the value of that element changes (from 0.3 to 0.6) and so does that of the first element (from 0.7 to 0.3). In this case, the improvement with regard to the third element does not lead to an increase in overall skill or performance value, since this improvement interferes negatively with the contributing value of the first element. When that element changes state, as it happens from Generation 2 and Generation 3, its value increases, but now the second element is maladjusted (its contributing value drops to 0.0; note that our model also allows for positive interferences).

Even incremental innovation, characterized by deliberately changing one component or constitutive action at a time, is therefore a very delicate matter; although one element may contribute positively to overall performance, there is a chance that it does do so at the price of lowering the contribution of several other elements. Consequently, for higher 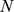 and 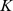, it gets increasingly harder for agents to find a configuration which outperforms its predecessor, and even small copy error rates may have large detrimental consequences. As a result, only very sizable populations will be lucky enough to contain an individual who does better than its parent.

Simulations proceeded as follows. We first generated *NK*-tables. An *NK*-table lists all string combinations for *N* elements, each with a corresponding, predefined contributing value (initially being drawn from a Uniform [0,1]; later we also considered Normal [0,1] and Gumbel [0,1] distributions). The string combinations with corresponding overall values on the right-hand side of Figure 2 could be interpreted as representing *part* of a predefined *NK*-table for 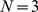, 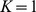. If an agent would try out a certain configuration, say *101*, its overall performance would be simply given by the average fitness value given in the *101*-row of the *NK*-table (i.e. 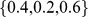 in the example). We generated 200 *NK*-tables for each combination 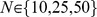 and 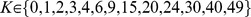, which amounts to 6,000 *NK*-tables in total.

Populations of size 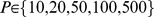 (and for a selected range of parameter settings of size 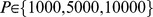; more on this in the Results section) had to "explore" these 6,000 *NK*-tables. Simulations were initialized at step 1 by assigning to each agent of Generation 1 the same string of size 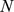, with a configuration and fitness randomly drawn from the *NK*-table under consideration. Each one of the next steps of the simulation consisted of the following sub-steps:

1. a new generation of 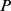 agents is introduced;
2. each individual of the new generation selects a cultural parent from the previous generation, and this depending on the parent's fitness;
3. the individual copies the selected parent's trait with copy error rate 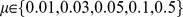;
4. each individual receives a fitness based on its acquired trait;
5. the new generation replaces the parent generation and the average and maximum fitness of the population, 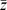 and 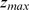, are measured.

Pay-off biases are thus implemented in the second sub-step. We considered two implementations, one in which parent selection is perfect, i.e. the single best parent is selected by each offspring individual (BEST); in the other, parents are selected proportionally to their fitness (WEIGHTED).

After 100 steps (i.e. after 100 generations), simulations were stopped, and three measures were computed. First, the maximum fitness of the last generation, or
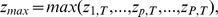



where 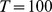 and refers to the last generation.

Second, the average cumulation between the first and later generations (as in [2]), or
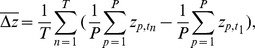



where 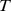 refers to the last generation, 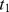 to the first generation, and 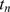 to the 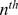 generation.

Finally, third, the cumulation between the first and last generation (as in [4]), given by
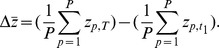



In order to compare the performance of populations of varying sizes, we applied, for each parameter combination, a Wilcoxon signed-rank test, comparing the sample of 200 observations (corresponding to the 200 *NK*-tables explored for each parameter combination) obtained for the populations under test. This pairwise Wilcoxon comparison is appropriate, since we let populations of varying sizes always explore the same *NK*-tables *and* let them start with identical initial strings/fitnesses.

Note that our model does not allow complexity and population size to evolve. This means we are able to compare only how populations of a fixed size are able to sustain a technology of a given complexity. Yet, we follow our benchmark studies [3], [4] here, and assume that from such comparisons can be inferred causal claims (i.e. claims about the extent to which demographic change may *favour* cultural change). Although we believe that this inferential step needs extra argument, we thus take it to be unproblematic here. Importantly, this does not undermine a negative result of our study: if it demonstrates the comparative advantage of larger over smaller populations to be non-robust, demographic explanations are compromised, regardless of whether or not the causal inference can be justified.

## Results

**Figure 3:**
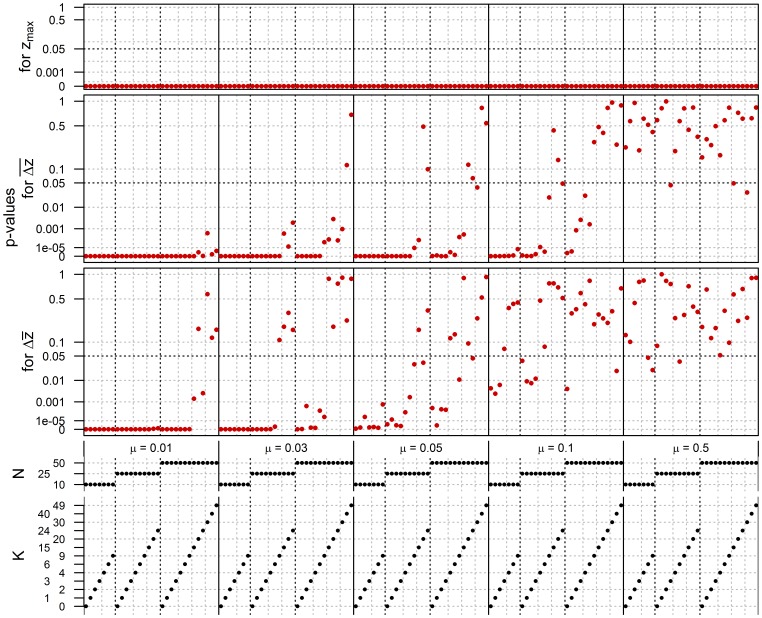
Comparison of *P* = 10 and *P* = 500, assuming Uniform distributions and WEIGHTED pay-off bias. Red dots indicate *p*-values from two-sided Wilcoxon signed-rank tests for 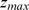 (upper), 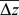 (middle) and 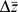 (lower), and this for parameter combinations marked by the black dots below.

Figure 3 compares populations of 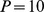 and 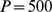, assuming Uniform distributions and WEIGHTED pay-off bias, with red dots plotting the *p*-values resulting from the two-sided Wilcoxon signed-rank tests for the parameter combinations marked by the black dots below. Here the null hypothesis is that populations of sizes 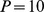 and 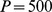 produce: no significantly different maximum fitness (upper part, *p*-values for 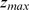); no significantly different average cumulation between the first and later generations (middle part, *p*-values for 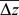); and no significantly different cumulation between the last and first generation (lower part, *p*-values for 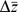). So red dots under the black dashed line indicate parameter combinations for which the null hypothesis should be rejected for a significance level of 0.05. For those combinations where we observed a significant difference, we checked whether it was in favour of 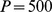 (so 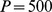 outperforming 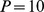) by means of one-sided tests. Since this turned out always to be the case, we do not explicitly refer to the results of these one-sided tests in the remainder.

The graphs exhibit several patterns. Let us start with the *p*-values for 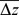 and 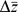. Generally, larger populations outperform smaller populations (so red dots fall under the 0.05 threshold) as long as complexity, expressed either as 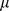, 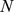, or 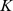 is low. When complexity increases, the larger populations of 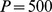 still produce higher maxima than the smaller populations of 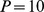 (as evident from the 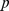-values for 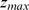), but the former lose their consistent advantage over the latter for two reasons. First, by lowering 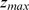-values, higher values of 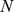 and/or 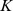 lead to *invisibility* of good parents, i.e. they contrast less with lower-skilled individuals. Consequently, given WEIGHTED pay-off bias, the contribution of inferior parents to transmission increases. Importantly, this holds for small and large populations alike. Second, higher values of 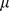 and 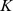 result in *instability*, in the following sense. For cumulation to occur, successive generations must be able to build on previous achievements; populations thus must be able to transmit a relatively stable knowledge base. That high values of 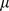 undermine this may be self-evident, but high values of 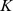 have a similar effect. Consider for instance the case where 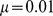 and 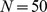. In this case, there is a 39.5% chance of at least one element changing state during transmission, an error which affects the fitness of 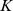 other elements. Now if 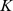 is high, say 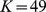, a good innovation is very easily lost; a transmission error in one element leads to 50 new draws, which, for a Uniform distribution, will average out close to 0.5. So even if the transmission error is beneficial (i.e. it leads to a higher contributing value for the element itself), it will be largely undone by the new values drawn for the element's interdependent elements. More colloquially, excellent traits can be expected to deteriorate dramatically in transmission if even one of their elements would change state.

Interestingly, although the qualitative results for 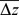 and 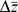 are the same, large populations are, in sustaining traits of higher complexity, slower to lose their advantage with respect to the former. An explanation for this is that the variance of 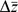 is larger than that of 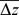 for 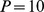, in particular in cases where a population effect is found for 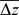 but not for 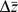. So, even if under these conditions the mean and median values of 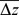 and 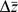 are similar, the variance of 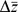 will be too sizable to yield a significant difference in the test for it. This evidently leaves the question why 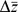 would exhibit a larger variance in the first place. Here the explanation is that, when 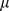 and/or 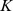 are sufficiently but not exceedingly high, small populations, due to their size, go through repetitive, quick episodes of substantial loss and cumulation. In case of 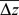, these fluctuations are averaged out by averaging over 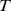, resulting in variances lower than those of 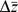.

Note that further increasing population size does not solve said issues of invisibility and instability, as can be gleaned from Table 1. That table gives the *p*-values of Wilcoxon signed-rank tests for 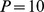 versus populations sized 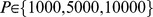, and this for the two first parameter combinations for which 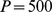 didn't outperform 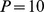. It appears that under these conditions even populations of 10,000 individuals are not significantly better at sustaining highly complex cultural traits than populations of only 10 individuals.

**Table 1:** *p*-values from Wilcoxon signed-rank tests assuming Uniform distributions, comparing 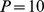 with 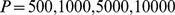, and this for the lowest parameter combinations for which 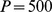 doesn't outperform 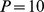.

	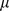	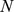	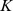	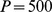	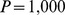	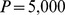	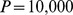
for 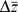	0.01	50	30	0.578356	0.700264	0.667116	0.592619
	0.01	50	40	0.12138	0.108733	0.12138	0.140984
for 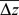	0.03	50	40	0.119341	0.118763	0.12524	0.119341
	0.03	50	49	0.691245	0.590933	0.578356	0.557679

Further, comparisons between populations of size 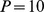 and populations of size 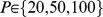 support the idea that size effects are transitive. That is, for these smaller population sizes, no effects were observed that were not also present when comparing 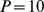 and 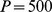.

Finally, trends observed for 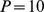 versus 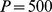 for Uniform distributions also occur under Normal [0,1] and Gumbel [0,1] distributions (see Figure S1 and Figure S2).

**Figure 4:**
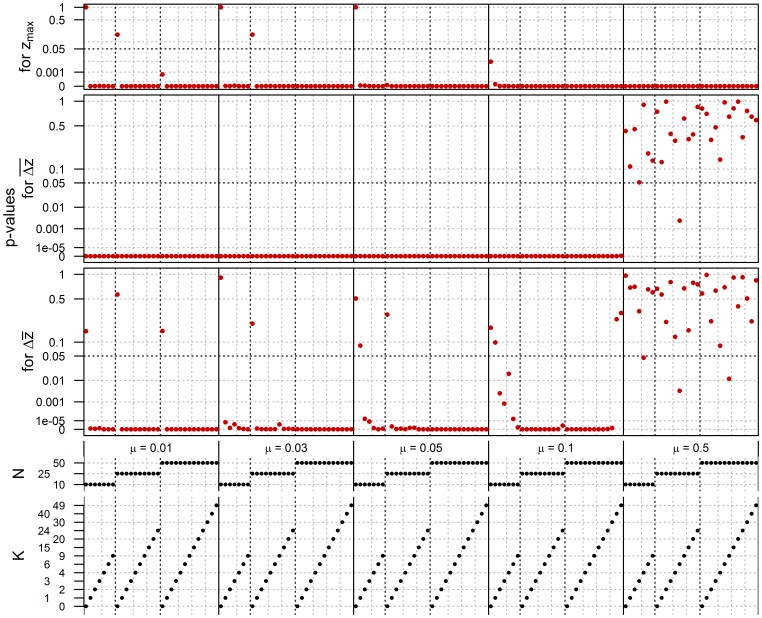
Comparison of *P* = 10 and *P* = 500, assuming Uniform distributions and BEST pay-off bias. Red dots indicate *p*-values from Wilcoxon signed-rank tests for 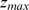 (upper), 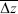 (middle) and 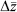 (lower), and this for parameter combinations marked by the black dots below.

Trends are different under the assumption of BEST pay-off bias, where offspring is able to identify and imitate the single best individual in the parent generation. As can be seen in Figure 4, demography now more generally makes a difference in sustaining cumulative culture. Only when transmission is highly erroneous (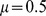) or 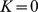, population size contributes little to accumulation.

Note that the results for BEST pay-off bias reinforce our earlier argument concerning invisibility. BEST pay-off bias by construction removes the invisibility constraint: however small the contrast between the best cultural parent and the lesser-skilled members of her generation, the BEST condition guarantees that she will be identified by all offspring. Under these circumstances, 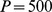 retains its advantage over 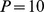 for higher 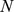
's and 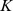
's, except when 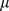 is high.

## Discussion

This study examined the robustness of a regularity suggested by previous modelling efforts, namely a strong dependence of cumulative culture on demography. More particularly, the aim was to verify whether that link was independent from—rather than an artifact of—previous models' assumptions about cumulation. To that effect, we added a measure of complexity to those already implemented in cultural-evolutionary models, and we adapted existing models so that cumulative cultural change could be expressed in terms of what we called 'S-complexity' (after Herbert Simon). This complexity is a function not only of a trait's number of components (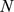), but also of the number of interactions between these components (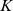). Our hypothesis was that in the face of increasing S-complexity, the link between demographic change and cumulative culture would collapse.

The results of the simulations reported here lend support to our hypothesis: except under the highly optimistic assumption of BEST pay-off bias, large populations tend to lose their advantage in sustaining cumulative cultural change when cultural traits get too intricate. We identified two reasons for this. The first is that high S-complexity weighs heavily on social learners' ability to stand out under WEIGHTED pay-off bias. That is, except when pay-off biased selection is perfect and offspring is able to identify the single best individual in the parent generation, offspring is very often imitating inferior parents whose pay-offs are insufficiently different from even the best individuals in the population. The second reason is that cumulative culture demands stability or continuity, which is undermined not only by high copy error rate, but also by high values of 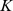. When 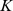 is high, even a slight change in a trait's set-up will have a profound impact on the trait's overall value. Thus, the slightest error in transmission has the potential to completely destroy a previous achievement; the latter may be haphazardly reinvented on a later date, but not due to a cumulative process of building improvements on improvements.

These results add to the suspicion that the dependence of cumulative culture on demography is not general, but applies to a specific range of cases (for empirical evidence questioning this dependence, [18], [19]; but see [20]). Previously, it has been shown to obtain only under a limited number of assumptions concerning learning biases [8], [21]; here it has been shown to obtain only insofar as previous assumptions about complexity are not violated and one makes the additional, highly optimistic assumption that naïve individuals are always able to identify and get the opportunity to learn from the single best parent in the population.

How does this bear on explanations of particular episodes of cultural change? Since assumptions about complexity couldn't be discounted by means of robustness analysis, the only option seems to attempt to discount them on empirical grounds. If it would turn out that the Upper Paleolithic transition (for instance) didn't correspond to increases of S-complexity, Powell et al's explanation [4] would stand firm. Conversely, it would compromise Powell et al's account if the transition were marked by the emergence of more intricate innovations, with increasing interdependencies among components (e.g., between procuring, transporting, preprocessing, and processing materials). We take it that as regards the Upper Paleolithic transition the choice between Powell et al's and our assumptions about complexity are underdetermined by the available evidence; so that currently neither their demographic explanation nor its negation can be discarded. So contrary to Powell et al's claims, it still may very well be that increased cognitive capacity (e.g., increased causal understanding of the interdependencies between components) gave rise to the Late Pleistocene emergence of modern human behaviour; or that some other factor or combination of factors made us modern.

More generally, this study shows the importance and usefulness of robustness analysis. Besides sorting out claims which hold independently from the simplifying assumptions of the models they are based on, robustness analysis usefully guides data gathering: it tells for which assumptions we still need empirical confirmation (i.e. those assumptions which it cannot discount) and for which we can remain blissfully, or at least safely, ignorant (i.e. those assumptions which are inessential to the phenomenon of interest). Robustness analysis therefore is and should be an integral part of model building and assessment.

## Supporting Information

Figure S1Comparison of *P* = 10 and *P* = 500, assuming Normal [0,1] distributions WEIGHTED pay-off bias. Red dots indicate *p*-values from Wilcoxon signed-rank tests for 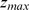 (upper), 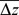 (middle) and 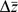 (lower), and this for parameter combinations marked by the black dots below.(TIFF)

Figure S2Comparison of *P* = 10 and *P* = 500, assuming Gumbel [0,1] distributions WEIGHTED pay-off bias. Red dots indicate *p*-values from Wilcoxon signed-rank tests for 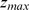 (upper), 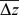 (middle) and 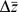 (lower), and this for parameter combinations marked by the black dots below.(TIFF)
