# Supplementary figures and images for: Complexity and Demographic Explanations of Cumulative Culture
Source: PLoS One. 2014 Jul 21;9(7):e102543. doi: 10.1371/journal.pone.0102543 (PMC4105626; doi:10.1371/journal.pone.0102543)

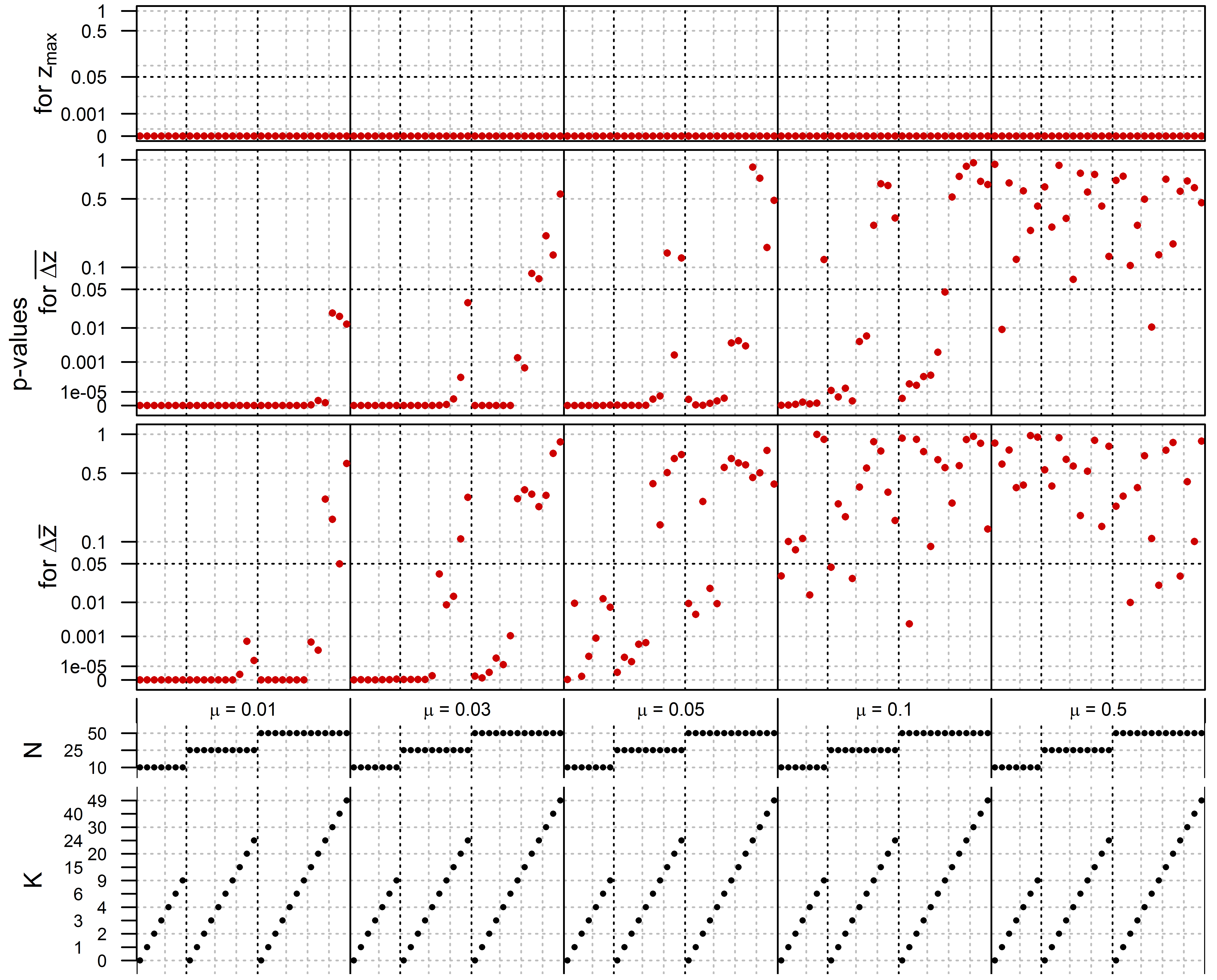

Supplement: Figure S1 — Comparison of P = 10 and P = 500, assuming Normal [0,1] distributions WEIGHTED pay-off bias. Red dots indicate p-values from Wilcoxon signed-rank tests for (upper), (middle) and (lower), and this for parameter combinations marked by the black dots below. (TIFF) [file pone.0102543.s001.tiff]

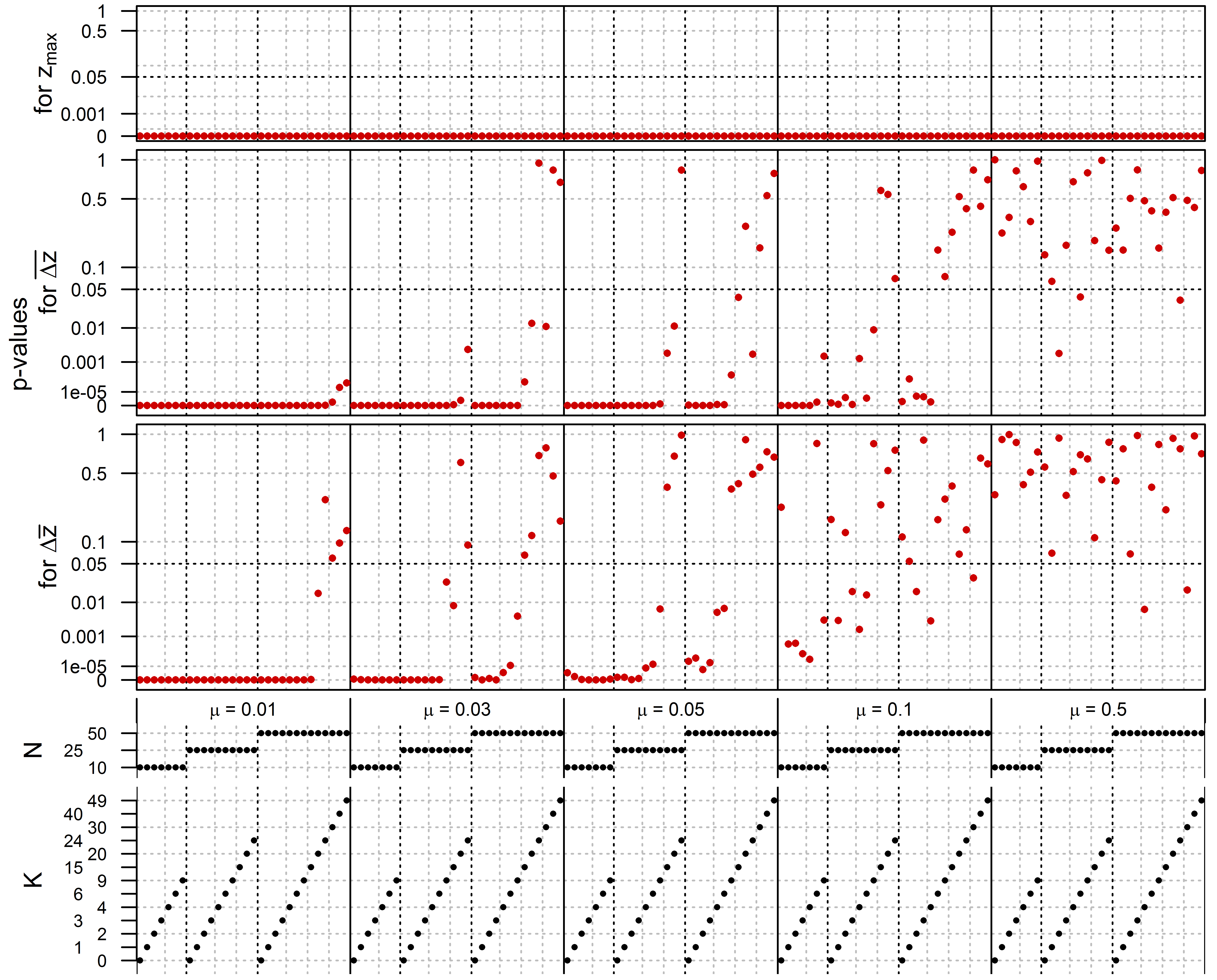

Supplement: Figure S2 — Comparison of P = 10 and P = 500, assuming Gumbel [0,1] distributions WEIGHTED pay-off bias. Red dots indicate p-values from Wilcoxon signed-rank tests for (upper), (middle) and (lower), and this for parameter combinations marked by the black dots below. (TIFF) [file pone.0102543.s002.tiff]
